# Exosome Traceability and Cell Source Dependence on Composition and Cell-Cell Cross Talk

**DOI:** 10.3390/ijms22105346

**Published:** 2021-05-19

**Authors:** Rabab N. Hamzah, Karrer M. Alghazali, Alexandru S. Biris, Robert J. Griffin

**Affiliations:** 1 Department of Radiation Oncology, University of Arkansas for Medical Sciences, Little Rock, AR 72205, USA; RNHamzah@uams.edu; 2 Center for Integrative Nanotechnology Sciences, University of Arkansas at Little Rock, Little Rock, AR 72204, USA; kmkadum@ualr.edu (K.M.A.); asbiris@ualr.edu (A.S.B.); 3 Nushores Biosciences LLC, Little Rock, AR 72211, USA

**Keywords:** exosomes, tumor cell-derived exosomes, normal cell-derived exosomes, nanoparticle (NPs), liquid biopsy

## Abstract

Exosomes are small vesicles with an average diameter of 100 nm that are produced by many, if not all, cell types. Exosome cargo includes lipids, proteins, and nucleic acids arranged specifically in the endosomes of donor cells. Exosomes can transfer the donor cell components to target cells and can affect cell signaling, proliferation, and differentiation. Important new information about exosomes’ remote communication with other cells is rapidly being accumulated. Recent data indicates that the results of this communication depend on the donor cell type and the environment of the host cell. In the field of cancer research, major questions remain, such as whether tumor cell exosomes are equally taken up by cancer cells and normal cells and whether exosomes secreted by normal cells are specifically taken up by other normal cells or also tumor cells. Furthermore, we do not know how exosome uptake is made selective, how we can trace exosome uptake selectivity, or what the most appropriate methods are to study exosome uptake and selectivity. This review will explain the effect of exosome source and the impact of the donor cell growth environment on tumor and normal cell interaction and communication. The review will also summarize the methods that have been used to label and trace exosomes to date.

## 1. Introduction

Exosomes are lipid bilayer nanovesicles released by all cells and biofluids in living organisms. They are very small (30–150 nm) and contain proteins, lipids, and RNAs [1,2]. Exosomes can reprogram target cells by delivering their contents to these cells. As a result, exosomes are being explored for use in the treatment and diagnosis of diseases [3], leading to increased interest in exosomal research [4,5]. Many researchers are working to understand exosomes’ role in cell communication, immune system regulation, and disease treatment and, most importantly, their effect on their environment [6]. Exosomes are released by all cells in the body, but their contents differ depending on their parental cells; this affects the exosomes’ function and selectivity to recipient cells [7].

Some work suggests that any exosome can be taken up by all cell types [8], while others show that exosomes selectively target certain cells [9]. For example, tumor cell-derived exosomes can be taken up by the same parent cells and not by other types of tumor cells and normal cells [10,11]. Neural cell-derived exosomes can also be taken up in a cell-specific manner [12]. This selectivity in exosome uptake can allow exosomes to be used in different applications. Exosomes can activate or suppress the immune system by regulating different immune cells [10]. Tumor cell-derived exosomes can stimulate tumor growth in both tumor and non-tumor environments [11]. Many works have sought to use exosomes as therapeutic vehicles to treat different types of diseases [13], such as breast cancer [14], lung cancer [15], prostatic cancer [16], pancreatic cancer [17], and brain cancer [18,19]. Additionally, Schwann cell-derived exosomes can improve axonal regeneration, while fibroblast-derived exosomes have no effect on axon regeneration [20].

While exosomes are promising for a variety of applications, tracking exosomes is very difficult due to their small size and heterogeneity. Prior efforts have been made to label exosomes with different types of dyes and visualize them with different imaging techniques, such as fluorescence confocal microscopy and high-resolution fluorescence microscopy [21,22]. Other groups have used nanomaterials to label exosomes and track them with computed tomography (CT), magnetic resonance imaging (MRI), and photoacoustic (PA) microscopy [23,24,25]. The latter has helped develop novel theranostic nanovesicles for potential use in the treatment of cancer. 

In this review, we will summarize the known relationships between donor and recipient cells in exosomal biology, as well as the labeling techniques that have been used to track exosome transit and the relative success or failure of these approaches.

## 2. Overview of Exosomes

### 2.1. Exosome Origins, Secretion Mechanics, and Structure

**Figure 1:**
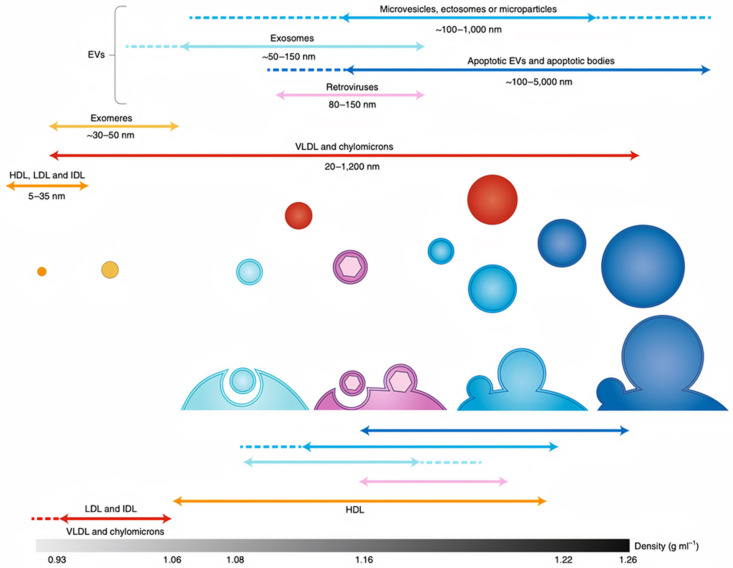
Size ranges of major types of membrane vesicles [33]. Figure reproduced with permission from Springer Nature.

**Figure 2:**
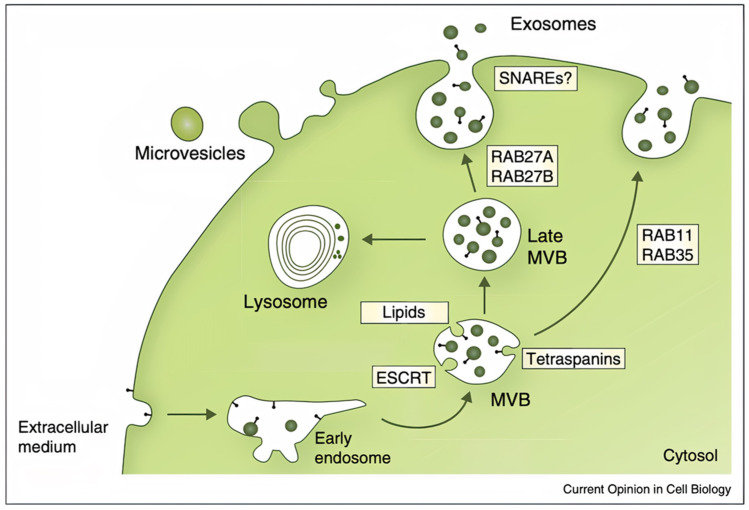
Exosome biogenesis and secretion [29]. Figure reproduced with permission from Elsevier.

Exosomes are nanostructural vesicles with a size range of 30–150 nm (Figure 1) that have gained increasing attention over the past few years [7]. They have endocytic origin, the endocytic compartment membrane, the plasma membrane, and cytosol contain all exosomal proteins. Exosomes have not been found to contain proteins of the mitochondria, nucleus, endoplasmic reticulum, or Golgi apparatus [26]. Exosomes originate from late endosomes, created by the inward budding of the limited multivesicular body (MVB) membrane. Within large MVBs, intraluminal vesicles (ILVs) are formed by the invagination of late endosomal membranes. When fused with the plasma membrane, ILVs are sent into the extracellular space, becoming exosomes (Figure 2) [27,28]. The proteins required to trigger exosome release are ESCRTs, RAB family, GTPase, and SNARE; these proteins are cell type dependent. Some cells require an ESCRT or RAB mechanism to release exosomes, while others do not [29,30].

Exosomes are surrounded by a lipid bilayer membrane (Figure 1) [31,32,33], which contains proteins and lipids adapted from the parent cells; most exosomal proteins are tetraspanin proteins [34]. In addition, there are different types of RNA in exosomes, including non-coding RNAs such as micro RNAs (miRNAs), as well as coding mRNAs, ribosomal RNA (rRNA), long noncoding RNAs (lncRNAs), and circular RNAs (circRNAs) [35]. Exosomes’ contents can be delivered to receptor cells by the interaction between exosomes and the target cells [35].

### 2.2. Known Exosome Functions

**Figure 3:**
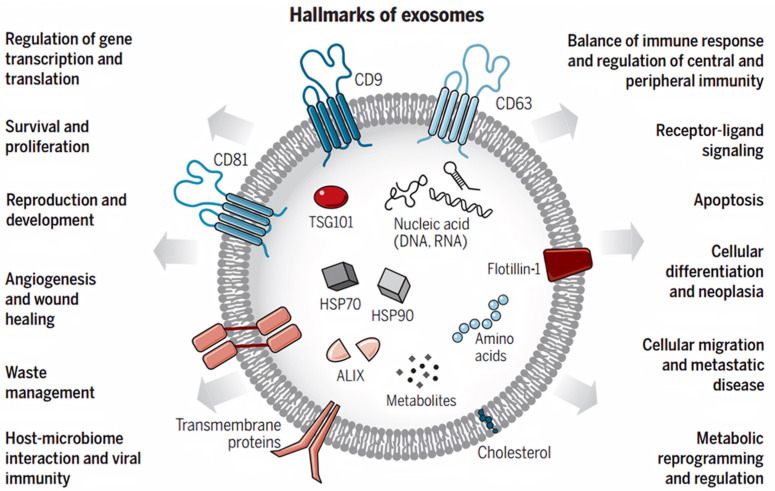
The proteins, lipids, and RNAs that exosomes bear and that have great impact on exosomes’ cellular communication and other functions [45]. Figure reproduced with permission from Science.

As we discussed in the previous section, exosomes possess in their structure various components originated from their parent cells, such as proteins, nucleic acids, and lipids. This unique structure makes exosomes excellent candidates to function as small intercellular communication molecules. Exosomes secreted from parent cells interact with recipient cells, influencing the behavior and phenotype features of the targeted cells [36]. As a result, exosomes can activate and/or suppress the immune system [7]. In addition, exosomes can be used as a biomarker of various diseases due to their specific protein and lipid profiles and the different types of RNA they carry, which can be similar to the original cell and its physiological state [37,38]. Additionally, as a delivery vehicle, exosomes can deliver drugs, RNAs, enzymes, and other therapeutic agents to the target cells to treat different types of diseases [17,38,39,40]. Furthermore, exosomes can be used in regenerative medicine to treat different types of central nervous system diseases; in such cases, the exosomes can mimic the artificial nanoparticles used in the field of regeneration medicine to develop novel treatments [41,42,43,44]. Figure 3 shows exosome compositions and functions.

### 2.3. Mechanism of Uptake

**Figure 4:**
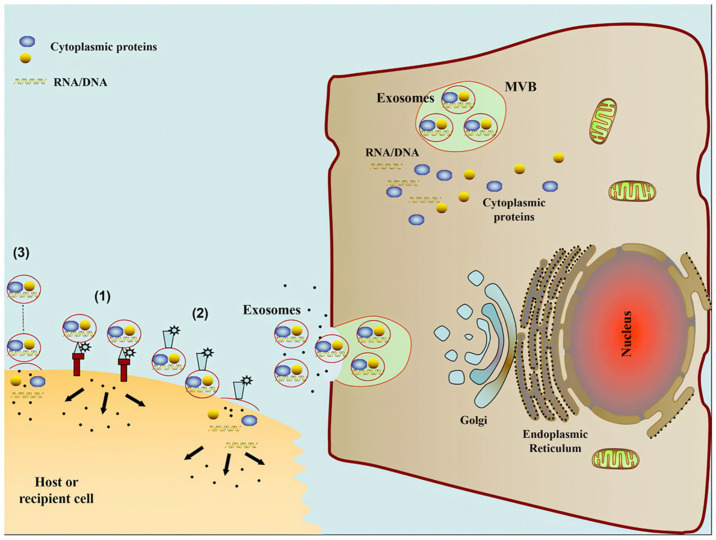
A schematic diagram of pathways involved in exosome-mediated cell-to-cell communication. (1) Exosomes signal recipient cells via direct surface-bound ligands. (2) Exosomes transfer activated receptors to recipient cells. (3) Exosomes may epigenetically reprogram recipient cells via delivery of functional proteins, lipids, and RNAs [7]. Figure and caption reproduced with permission from Springer Nature.

As the field learns more about exosomes’ integral role in biological functions and diseases, as well as their theranostic uses, understanding how they communicate with and are taken up by cells is becoming crucial [46]. An exosome’s cargo has been proven to affect target cells in three different ways: (1) Trigger the target cells directly by surface-bound ligands; (2) carry activated receptors to the target cells; and (3) epigenetically reprogram target cells by delivering their proteins, RNAs, and lipids (Figure 4) [7]. The way in which exosomes are taken up is influenced by protein interactions, which facilitate endocytosis [47]. Exosomes adhere to the membrane of target cells due to the abundance of tetraspanins on their membrane; CD81 and CD9 are tetraspanins that contribute to phagocyte fusion [47,48]. In addition, integrins and immunoglobulins are involved in a variety of functions between target cells and exosomes, such as cell signaling, cell-to-cell adhesion, antigen presentation, and leukocyte transendothelial transmigration [49]. The mechanism of uptake for exosomes is exosome-source dependent. More studies are needed to help researchers and physicians understand of the role of exosome source-cell on recipient-cell internalization. 

### 2.4. Exosomal Applications

The small size of exosomes and their components makes them very interesting for use in different biomedical applications, such as drug and nucleic acid delivery, biomarker molecules, anticancer vaccines, and regenerative medicine. For example, several studies have used exosomes for drug and nucleic acid delivery, reporting that exosomes can deliver various types of cancer drugs such as paclitaxel and doxorubicin [50,51]. In one study, doxorubicin was loaded into exosomes, leading to quick cellular uptake and redistribution of the drug from endosomes to the cytoplasm and nucleus. Unlike doxorubicin alone and doxorubicin-incorporated liposomes, exosomes do not accumulate in the heart; as a result, using exosomes to deliver doxorubicin could potentially reduce cardiac side effects and enhance the therapeutic index of the drug [52]. 

The use of exosomes as biomarker molecules was reported by Lin et al. [53], who showed that this can reduce the need for invasive surgeries to monitor diseases. For example, exosomes from serum samples have been utilized as biomarkers in lung, pancreatic, and ovarian cancers [54,55,56]. Additionally, the antigens on the surface of exosomes shield against specific types of cancer, making exosomes ideal candidates for cancer vaccines [57]. In addition, exosomes have been widely used in tissue regeneration due to their effect on cell phenotype, proliferation, and differentiation [58]. 

Exosomes are also being exploited in liquid biopsies for cancer diagnosis. Liquid biopsy enables a molecular profile of biofluids. This approach is popular because it is non-invasive and less expensive than the traditional tissue biopsy [59]. Liquid biopsies for cancer fall into three main classes: Circulating tumor cells (CTCs), circulating tumor DNA (ctDNA), and exosomes [60]. Exosomes are excellent targets for liquid biopsy due to their unique features, such as release by living cells and possession of biological information from parent cells, which is opposite to the cell-free DNA (cfDNA) secreted through apoptosis or necrosis [61]. In addition, most cfDNA from human plasma is located in exosomes [62], and researchers found that plasma or a peripheral blood sample from patients with advanced serous epithelial ovarian cancer has more mitochondrial DNA copies in exosomes than in cfDNA [63]. Moreover, exosomal DNA has plenty of biological information and shows higher sensitivity for prognosis prediction than cfDNA. Researchers have used a combination of exosomal RNA mutation and cfDNA to increase the accuracy of mutation detection [64]. In addition, unlike CTCs, exosomes can be obtained from any body fluids and can be isolated by methods that are easier than CTC isolation [65]. Furthermore, exosomes have high biological stability, which is a major advantage for clinical applications [66]. Not surprisingly, the first commercial test for liquid biopsy based on exosomes was produced in 2016, named the ExoDx Prostate (IntelliScore) (EPI) test. It is a non-invasive, urine-based test for prostate cancer diagnosis. With even greater understanding of exosome secretion and uptake patterns, more commercialized kits will no doubt be available very soon.

**Scheme 1:**
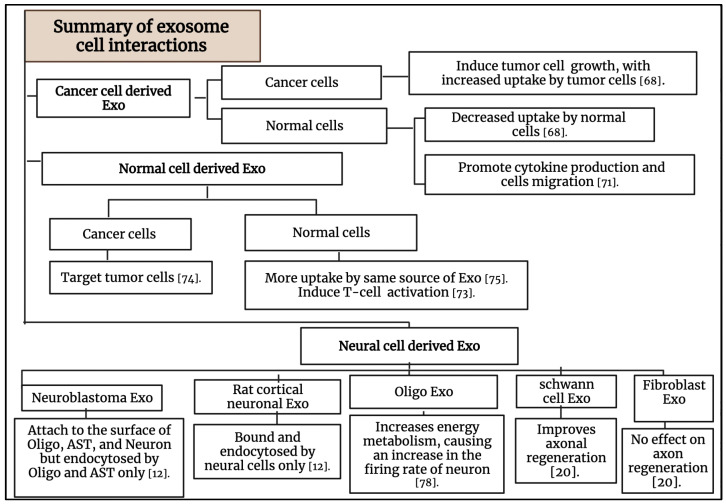
Summary of exosome-cell interactions.

Despite the variety of applications for exosomes, researchers still do not fully understand the effect of exosome source on their actions and therapeutic effect. In the next sections and Scheme 1, we will summarize what is currently known about the effect of exosome source on exosome-cell interactions and communication. 

## 3. Exosome-Cell Interactions

### 3.1. The Interaction of Cancer Cell-Derived Exosomes with Tumor Cells and Normal Cells

**Figure 5:**
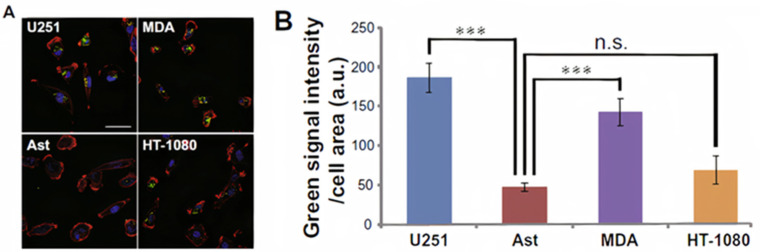
Effective uptake of U251-MG (U251) cell-derived exosomes (U251exo) by their parental cells and other cancer cell lines. (**A**) U251 cells, MDA-MB-231 (MDA) cells, HT-1080 cells, and AST were treated with U251exo for 12 h (bar 50 μm). (**B**) Green fluorescence intensity per cell area was determined by image analysis [68]. Figure and caption reproduced with permission from Elsevier.

Cancer cell-derived exosomes (CDE) have been proven to have several functions in tumor progression. However, the actual mechanism following their transportation is not clear yet [67]. One study investigated the cellular uptake of the human exosome U251exo by different types of tumor cells and astrocyte cells [68]. Researchers have demonstrated that U251exo were taken up by human brain cancer cells (U251-MG), human breast cancer cells (MDA-MB-231), and human fibrosarcoma cells (HT-1080), as well as, to a lesser extent, by human astrocytes cells, as shown in Figure 5. The protein ligands were independent in this type of communication, while the lipid components were dependent. U251exo was highly enriched with phosphatidylethanolamine, a unique lipid component of U251exo that facilitated its interaction with the U251, MDA, and HT among astrocytes.

**Figure 6:**
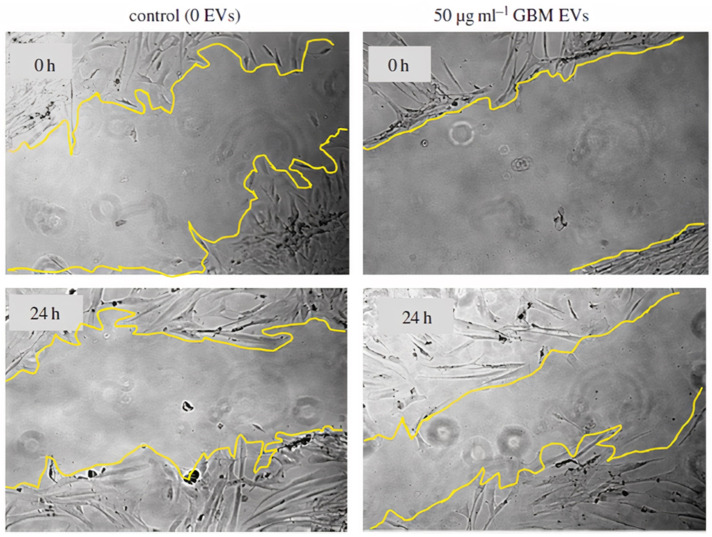
Glioblastoma EVs promote normal astrocyte migration [71]. Figure reproduced with permission from Royal Society.

CDE can affect both their immediate environments and distant targets; they can even promote tumor progression by changing non-cancerous cells around the tumor microenvironment [69]. CDE can also send anti or pro-tumor signals to immune and nonimmune cells, thereby altering their functions. The ways in which CDEs reprogram cells include uptake by the recipient cells and/or cell surface signaling [69]. Several studies have explained that tumor extracellular vesicles (EVs) enhance tumor cell migration but hamper lymphocyte migration [69,70]. Other researchers have demonstrated that human glioblastoma-derived exosomes can interact with normal human astrocyte cells and change their local environment by promoting cytokine production and astrocyte cell migration (Figure 6), causing tumor-like phenotypic changes in the normal astrocyte cell population [71]. 

### 3.2. The Interaction of Normal Cell-Derived Exosomes with Normal Cells

Exosomes secreted by immune cells could be crucial for communication between cells [72]. Several studies have been done to determine the effect of exosome source on cell-cell communication and immune response [6,9]. Herein, we will describe the effect of exosomes derived from immune cells on immune cell stimulation. In addition, we will explain the effect of exosome source on their uptake by immune cells. Exosomes derived from human dendritic cells (DCs) carry functional MHC class I and class II molecules that can be used to activate T-cells, thus increasing interest in using DC-derived exosomes in cancer clinical trials [73,74]. Researchers have isolated exosomes from live human primary DCs and studied their impact on CD4+T lymphocytes in vitro. One study showed that DC-derived exosomes induce T-cell activation by inducing the release of Th1 cytokine IFN-γ. First, the researchers tested the uptake of exosomes by T-cells. Then, they monitored the activation of T-cells by measuring the up-regulation of the early T-cell activation marker CD69. In both studies, they compared the exosomes with other types of EVs. Inducing IFN-γ secretion by Th lymphocytes is a sign of immune response that is expected to be effective in anti-tumor immunotherapy [73]. In another study, researchers proved the selectivity of exosomes to the target cells based on their origin by adding mesenchymal stem cell (MSC)-derived exosomes to MSCs and monocyte cells. The MSC-derived exosomes were taken up by MSCs rather than monocyte cells [75]. These studies confirm that exosome-cell interaction is source dependent. This is a very important feature that makes exosomes suitable bio-nano-agents for targeting and treating different diseases. 

### 3.3. The Interaction of Normal Cell-Derived Exosomes with Tumor Cells

**Figure 7:**
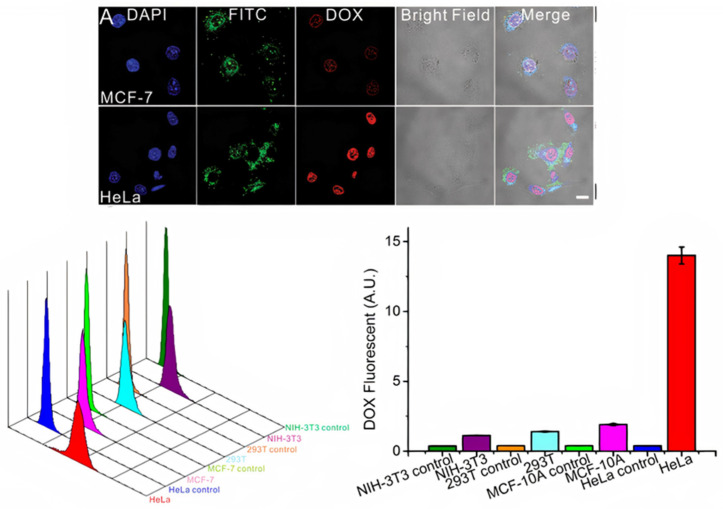
Top: Confocal microscopy images of the cellular uptake of fluorescently labeled FA-AuNR@RGD-DOX-Exos. MCF-7 (ανβ3+ and FR+) cells and HeLa (ανβ3+ and FR+) cells were treated with fluorescently labeled macrophage exosomes for 4 h. Bottom: Flow cytometric profiles and mean fluorescence intensity of the normal cell lines NIH-3T3, 293T, and MCF-10A and cancer cell line HeLa upon incubation with macrophage exosomes [74]. Figure and caption reproduced with permission from Wiley.

Multiple studies have proven that macrophage-derived exosomes can keep the topology of plasma membrane proteins and evade being taken in by mononuclear phagocytes, which is important in the treatment of tumors [74]. Macrophage-derived exosomes have been utilized to target tumor cells. Researchers studied the effect of the integrin protein on exosome-cell interaction by delivering drug-loaded exosomes to different types of tumor cells, such as HeLa and MCF7, and normal (non-tumor) cells (NIH-3T3, 293T, and MCF-10A). The macrophage-derived exosomes accumulated on the HeLa cells, which highly express ανβ3+ and FR+, more than the other tumor cell type (MCF7 are ανβ3- and FR-) and the normal cells Figure 7 [74]. Additionally, tumor cells’ uptake of exosomes derived from human placental MSCs was investigated, and the results showed that exosome uptake was selective depending on their source due to the exosomal envelope that maintains the identity and compatibility of transference. This research proved that MSC exosomes are taken up more by MSCs than by tumor cells. Exosomes released from the same cell type share a certain signature, which is also found in their parent cells [75].

### 3.4. The Interaction between Exosomes and the Nervous System

**Figure 8:**
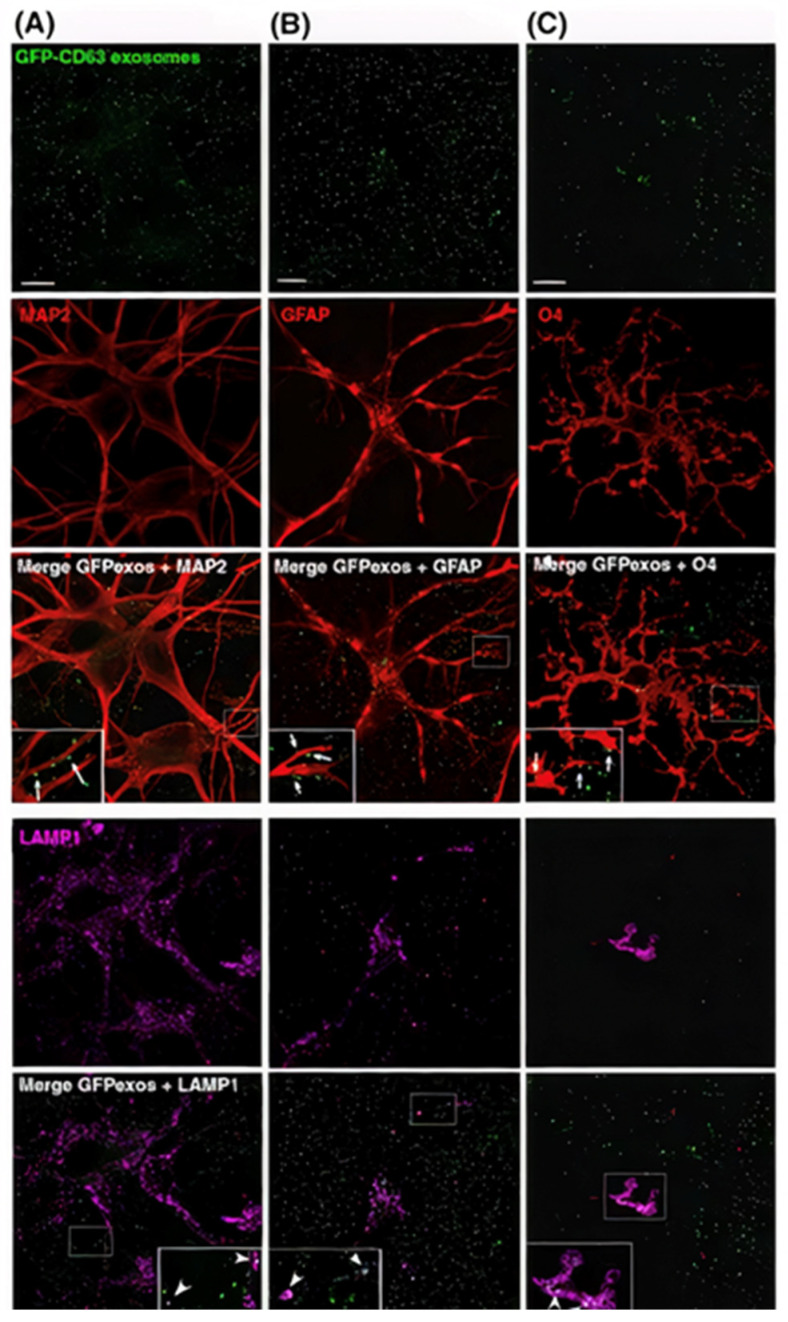

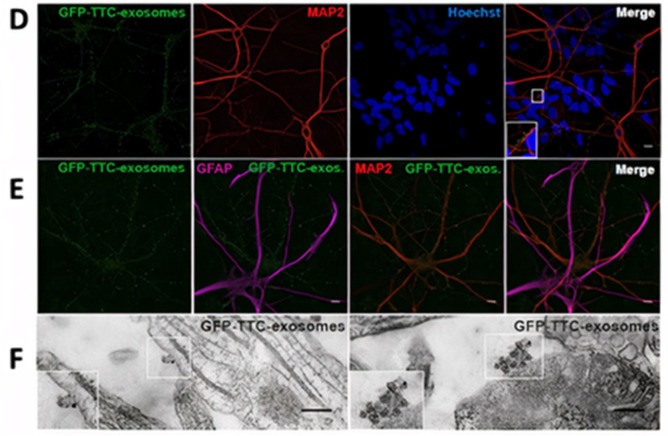
Neuroblastoma exosomes carrying GFP-CD63 bind to and are endocytosed by neurons and glial cells. (**A**) MAP2, (**B**) GFAP, and (**C**) O4 to label neurons, astrocytes, and oligodendrocytes, respectively, and with antibodies against Lamp-1 (**A**–**C**) to stain late endosomes and lysosomes. (**D**–**F**) Neuronal exosomes bearing GFP_TTC bind specifically to neurons [12]. Figure and caption reproduced with permission from John Wiley and Sons.

It is believed that exosomes secreted by neural cells play a major role in neurodegenerative diseases [76]. Several studies proved that exosomes can regulate neuroinflammation, significantly impact the treatment of particular neurological diseases, and promote neurogenesis and neurogenic physiological location [76,77]. Exosomes from nerve cells are also specific in their intracellular communications. Researchers showed that neuroblastoma exosomes bind randomly to rat hippocampal cells and glial cells and could only be endocytosed by glial cells (Figure 8A–C). Briefly, GFP-CD63 exosomes purified from N2a^GFP-CD63^ cells were incubated with hippocampal cells (14 DIV) stained with anti-MAP2 to detect neurons, anti-GFAP to detect astrocytes, anti-O4 to detect oligodendrocytes, and anti-Lamp-1 to stain the late endosomes. Neuroblastoma exosomes attached to the surface of neurons, astrocytes, and oligodendrocytes without any specificity. While the anti-Lamp-1 co-staining showed the neuroblastoma exosomes inside Lamp-1-positive late endosomes of oligodendrocytes or astrocytes, the exosomes were not present within Lamp-1-positive compartments of neurons. In contrast, stimulated rat cortical neuronal exosomes were bound and endocytosed by neurons only, as explained in Figure 8D–F. In this part, GFP_TTC exosomes released by rat cortical neurons over synaptic activation were added to rat hippocampal cells (14 DIV) stained with anti-MAP2 to detect neurons and anti-GFAP to detect astrocytes. The exosomes strongly attached to MAP2-positive soma and dendrites and did not attach to MAP2-negative cells and GFAP-positive astrocytes. Rat hippocampal neurons incubated with rat cortical neurons were immunogold-stained against GFP to show the presence of single or aggregated exosomes on the surface of the neuron [12]. It is clear from these studies that neuronal cells can uptake different types of exosomes, but the effect of this uptake on neuron physiology is still under investigation.

**Figure 9:**
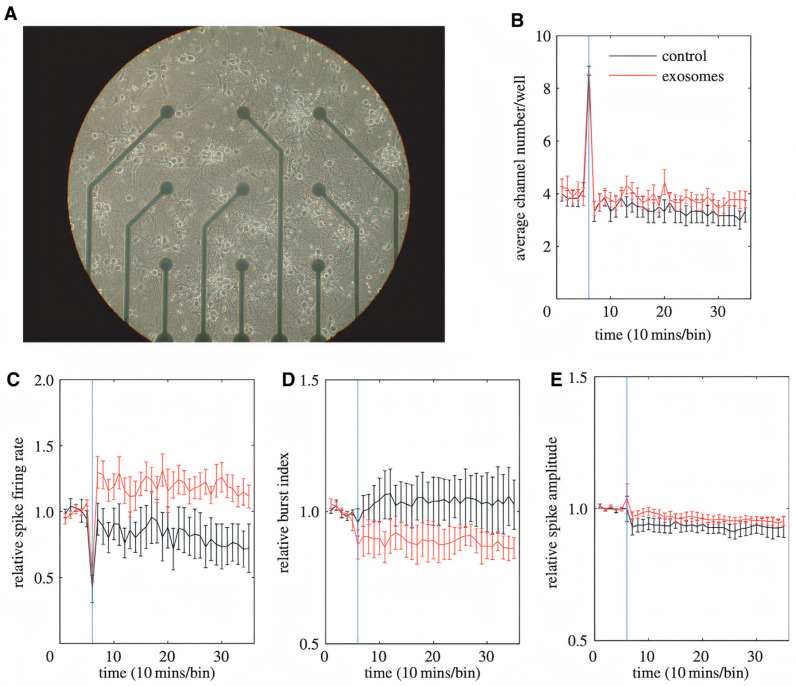
Impact of oligodendroglial exosomes on neuronal electrical activity. (**A**) Image of a 14 DIV pCN culture grown in a 9-channel MEA. Electrode spacing is 200 mm. (**B**–**E**) Recordings were performed at DIV 14. The blue vertical line indicates the application of pOL exosomes (red graph, n ¼ 9) or sham control (black graph, n ¼ 6) after 1 h of recording. Average channel number/well (**B**), relative spike firing rate (**C**), relative burst index (**D**), and relative spike amplitude (**E**) are depicted over 6 h of recording. Error bars+s.d. (* *p*, 0.05; Student’s *t*-test) [78]. Figure and caption reproduced with permission from Royal Society.

A study by Fröhlich et al. showed that administration of mouse oligodendroglial derived exosomes to mouse primary cortical neurons improves their action potential firing rate, modifies cellular signal transduction pathways, and alters their transcriptome. The researchers cultured a mouse primary cortical neuron on MEA chips (six wells, nine electrodes per well) for 14 days to create a circuit of neurons and cause spontaneous electrical activity. One hour after recording started, primary oligodendroglial-derived exosomes were added. The recording showed that after the exosomes were added, the relative spike amplitude did not change, but the relative firing rate instantly significantly improved (Figure 9c) and the relative burst index was moderately reduced (Figure 9d). These results confirm that oligodendrocyte-derived exosomes keep up the energy metabolism of neurons, causing an increase in the firing rate. These results also confirmed the finding about microglia-derived EVs working at the presynaptic site of the excitatory synapse by improving the release probability of synaptic vesicles throughout the metabolism induction of sphingolipids [78].

**Figure 10:**
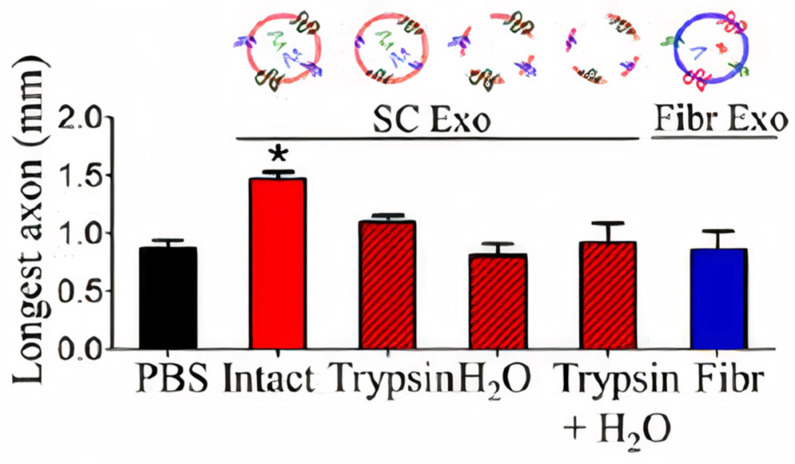
Effect of pre-treated exosomes from SCs and intact exosomes from fibroblasts (Fibr) on axonal regeneration three days after axonal damage [20]. Figure and caption reproduced with permission from John Wiley and Sons.

Previous work explained the effect of exosomes on neuron physiology and how exosomes increase the firing rate of neurons. Research has also shown how other sources of exosomes influence regeneration in the peripheral nervous system, as reported by Lopez-Verrilli et al. In this work, the researchers studied the effect of exosome source on axon regeneration. This was proved by adding exosomes derived from Schwann cells and fibroblast cells to dorsal root ganglia cells (DRGs). Exosomes derived from Schwann cells improved axonal regeneration through a local working on axons and inhibited RhoA and GTPase activation in growth cones (Figure 10). In contrast, exosomes derived from fibroblasts had no effect on axon regeneration. This study demonstrated that the content and composition of exosomes should be cell-specific to improve axonal regeneration [20].

## 4. Exosome Labeling and Tracking: Bridging the Gap to Understand Exosome Functionality

**Figure 11:**
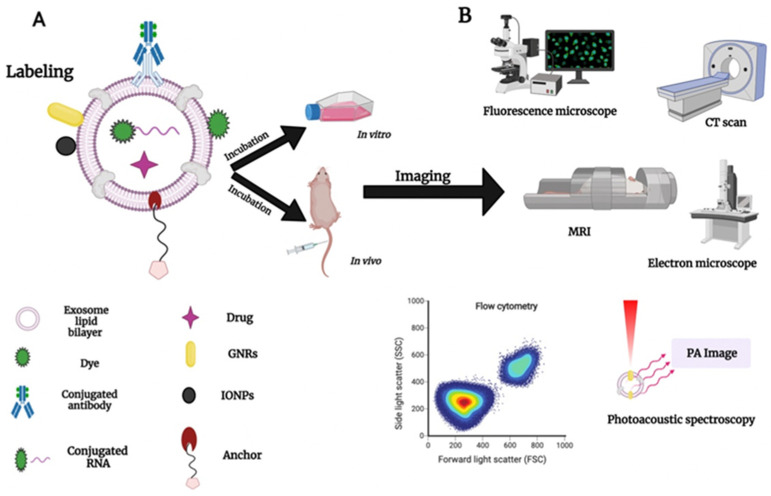
Illustration of labeling exosomes with different probes and NPs (**A**), and different instruments that can be used to image exosomes in vitro and in vivo (**B**). Here, fluorescence microscopy is used as an example for all types of fluorescence-based imaging technique. In addition, a number of other imaging modalities can be employed when exosomes have proper nanomaterial labeling. GNRs: Gold nanorod; IONPs: Iron oxide nanoparticles.

Understanding exosomes’ transportation or movement within the biological system will provide the scientific community with a clear visualization of exosomes’ functionality, and exosomes cell-specific uptake selectivity. The most challenging aspects of exosomes are their small size and heterogeneity, which make them hard to label and track. Several researchers have used different types of lipophilic dyes, such as the PKH family, DiL-family, and FM-family, to stain the lipid membrane of exosomes to make it suitable for tracking and visualization [79,80,81]. Nanoparticles (NPs) have also been widely used in biomedical applications [82,83]. Incorporation of artificial nanoparticles with exosomes might improve the treatment and diagnosis of a variety of diseases by developing a new natural nano-agent that is suitable for imaging. So far, few studies have been done to incorporate exosomes with artificial nanoparticles to develop a theranostic nano-agent [23,84]. María et al. used hollow gold nanoparticles (HGNs) to label exosomes. They found that incubating recipient cells with exosomes labeled with HGNs decrease the cell viability after irradiation. This study confirms the benefit of incorporation of NPs within exosomes to develop new optical hyperthermia therapies [75]. A variety of methods, including electroporation, sonication, chemical conjugation, and transfection have been used to label and/or load exosomes with drugs, nucleic acids, and NPs. These methods are still being optimized and need more work to prove that they do not affect exosome integrity and functionality. Exosomes membrane anchoring is a suitable surface modification strategy to assist cellular uptake. It depends on using lipids or peptide components [85]. Changsun et al. developed a new surface modification system for exosome that consists of an anchor, spacer, ligand, and doxorubicin. The cell anchor used as a fluorescent lipophilic boron-dipyrromethene (BODIPY), which can be inserted into the exosome membrane due to its hydrophobicity/lipophilicity. Poly (ethylene glycol) (PEG) was used as a spacer to conjugate the BODIPY-anchors, to help increase exosomes’ half-life and prevent exosomes aggregation at higher concentrations. Arg-Gly-Asp (RGD) peptide was used as a specific targeting ligand for integrin, which is overexpressed in melanoma. This system exhibits active targeting of B16F10 cells, more cellular uptake, and specific target accumulation leading to tumor growth inhibition in vitro and in vivo [86]. Other research used CP05 peptide specified as phage display for targeting, capturing, and cargo loading for exosomes by binding to the CD63, a well-known exosomal protein marker [87]. The results suggest that specific targeting peptides on exosomes can be achieved by CP05 anchoring, resulting in distribution of exosomes to specific tissues in vivo. Interestingly, CP05 anchoring can capture exosomes from patient’s serum, at a level that was 54% of the amount of exosomes captured by ultracentrifugation [87]. Thus, the peptide anchor strategy is a beneficial engineering approach that can be used as a tool for exosome capturing, drug delivery targeting, and investigation of gene function in vivo. Figure 11A shows the latest exosome labeling approaches with multiple probes and NPs.

Different instruments have been used to image and track exosomes within target cells in vitro and in vivo, such as regular confocal fluorescence microscopy [21], high resolution fluorescence microscopy such as STED [22] and PALM/Storm [80], scanning and transmission electron microscopy (SEM and TEM) [88], PA microscopy [23], MRI [24], flow cytometry (FC) [81], and CT scan [25] as shown in Figure 11B. Some of these instruments track exosomes by staining their membrane, such as confocal microscopy, or by incorporating other artificial nanoparticles, such as PA, MRI, and CT. More work needs to be done to understand the accuracy of each instrument in tracking and visualization of exosomes in vitro and in vivo and which instrument can be used when we translate exosome application to the clinic.

## 5. Conclusions

In general, exosome biogenesis, secretion, and functions are known, and exosomes have been proven to be involved in many biological processes. Exosomes have unique functions depending on their original cell type, allowing them to be used as drug delivery vehicles, therapeutic nano-agents, and biomarkers or diagnostic tools. Research has proven that exosomes derived from tumor cells suppress the immune cells and support cancer metastasis, in a cell type dependent fashion. Uptake by cells also depends on the exosomes’ original cells. In addition, exosomes can be used to treat different conditions of the nervous system. Several studies have proven that the interaction between nerve cells and exosomes is critical for CNS function and development and highly cell type dependent.

Further studies should be established to understand what cells work best to develop specific nano-drug carriers and biomarkers based on exosomes and the exosomes’ source, and whether loading exosomes changes behavior; labeling strategies need to be further optimized. Knowing the exosome’s dose and the half-life is important in order to use exosomes as diagnosis and treatment vesicles, a method that is more reliable for quantifying labeled exosomes.

In addition to these questions, the best way to track exosomes and determine their final destination is still under investigation. Exosomes’ heterogeneity and small size make them difficult to track. The vast number of circulating ‘normal’ exosomes in the blood stream present a great challenge in deciphering some of these more refined questions about vesicles secreted by pathological tissues. Therefore, developing a reliable technique to track exosomes loaded with drugs, RNA, and NPs in vitro and in vivo will help us understand more about the biology of these vesicles, leading to more accurate information about their functions. In turn, this knowledge will help us translate exosomes from the lab to the clinic and use them as a new source of information to treat a variety of diseases and monitor the overall health of an organism.

## Data Availability

Not applicable.

## Abbreviation

NPS	Nanoparticles
CT	Computed tomography
MRI	Magnetic resonance imaging
PA	Photoacoustic microscopy
MVB	Multivesicular body membrane
ILVs	Intraluminal vesicles
ctDNA	Circulating tumor DNA
CTCs	Circulating tumor cells
cfDNA	Cell-free DNA
Exo	Exosomes
AST	Astrocyte cells
Oligo	Oligodendrocytes cells
CDE	Cancer cell-derived exosomes
U251-MG	Human brain cancer cells
MDA-MB-231	Human breast cancer cells
HT-1080	Human fibrosarcoma cells
EVs	Extracellular vesicles
DCs	Dendritic cells
MSC	Mesenchymal stem cell
14DIV	Hippocampal cells
DRGs	Dorsal root ganglia cells
SCs	Schwann cells
HGNs	Hollow gold nanoparticle
BODIPY	Boron-dipyrromethene
PEG	Poly ethylene glycol
RGD	Arg-Gly-Asp
SEM	Scanning electron microscopy
TEM	Transmission electron microscopy
FC	Flow cytometry
GNRs	Gold nanorods
IONPs	Iron oxide nanoparticles
